# Digitalis1A paper read before the Veterinary Medical Association of New York County, January 4, 1899.

**Published:** 1899-02

**Authors:** H. D. Hanson

**Affiliations:** New York City


### THE JOURNAL
OF
### COMPARATIVE MEDICINE AND VETERINARY ARCHIVES.

Vol. XX. *FEBRUARY, 1899.* No. 2.

### DIGITALIS.[1]

By Prof. H. D. Hanson, D.V.S.,
NEW YORK CITY.

We learn by repetition. With this in view, I have selected digitalis as my subject, in order to refresh your memories upon the actions and indications for use of the drug, and also to emphasize certain misuses.

Before speaking of digitalis it may be well to call your attention to the classification of remedies in order to know under which head this drug belongs.

Remedies may be classified as medicinal, non-medicinal, or disinfectant.

Medicines have to enter the circulation in a state of solution before their action is obtained; non-medicinal agents act locally without absorption into the blood; disinfectants destroy or check agents producing disease.

Medicines are either functional medicines or organic medicines.

Functional medicines, also called symptom medicines, affect the function of an organ, but do not produce any alteration in structure that can be recognized after death. Examples are: Belladonna, opium, digitalis, aconite, aloes, tannin, etc.

Organic or disease medicines act upon or so change the tissues of the body as to alter the system by their use, as iron, cod-liver oil, phosphates, iodine, mercury, etc.

Functional medicines are given for certain symptoms, as pain, and their action is usually secured by one dose, while organic medicines are given for the disease itself, and have to be given for some time in order to obtain the desired effect.

[1] A paper read before the Veterinary Medical Association of New York County, January 4, 1899.

Functional medicines include neurotics, eliminatives, and astringents, while organic medicines include restoratives and alteratives.

Neurotics are classified under three heads: 1. Those called narcotics, which are both stimulants and sedatives, as alcohol, opium, belladonna, cannabis indica. 2. Those which stimulate certain nerve functions, as digitalis, ammonia, strychnine, camphor, ergot, etc. 3. Those acting as sedatives to certain nerve functions, as aconite, tartar emetic, cocaine, etc.

Neurotics are those medicines acting on the functions of the nervous system, and produce special symptoms. Any one drug of this class does not, and cannot, control all the functions of the nervous system, as these functions are too numerous and altogether different to be controlled by one neurotic. These medicines do not cure disease, but only relieve symptoms.

Digitalis, commonly called foxglove, is included under the second division of neurotics, namely, among those that stimulate only the nerve functions. The leaves are the parts mostly used, and are obtained from the two-year-old plant, *Digitalis purpurea*. The leaves have a bitter taste, are odorless in the recent state, but have a faint narcotic odor when dried. Beside containing the active principle of which digitalin is prominent, it contains a volatile oil, a fatty matter, coloring matter, albumin, starch, sugar, gum, and salts of potassium and calcium.

Experimentally upon animals, it has been observed that digitalis has its important action upon the circulatory apparatus, increasing the contractile power of the heart, and also the muscular coats of the arteries through the nerve ganglions at the branches or divisions of the vasomotor nerves going to the arteries.

There is a rise in arterial pressure by its action on the muscular fibres, by the increase of the heart's action, and by the vasomotor spasm. The diastole is prolonged, the systolic power is increased, causing an increased amount of work under its influence. The peripheral ends of the inhibitory nerves, as well as the cardiac, are stimulated.

In repeated medicinal doses, the pauses between the pulse beats become longer, the individual beat slower, longer, fuller, and stronger, showing that the heart is acting with more force than normal.

Digitalis is a cardiac stimulant, and is given to produce or increase the contractile power of the heart's muscle and help it to regain its normal size. Its action is principally on the ventricles, and does not cause all of the fibres to act equally, and thus a streaked appearance is shown, the fibres affected becoming paler in color. Digitalis increases the systole of the heart at the expense of the diastole.

Large and toxic doses produce nausea and vomiting (in those animals that vomit), muscular weakness, cold sweat, irregular heart, diuresis, diarrhœa, labored breathing, extreme prostration, a rise in blood-pressure soon followed by a lowering of the same, stupor or delirium, convulsions, and death.

The change of position causes a change in the pulse. If recumbent and quiet, the pulse may be slow and strong, while excitement or change of position causes the pulse to become rapid, irregular, small, and feeble.

There is an over-balancing of the systolic irritation over the diastolic stimulation, causing the pulse to become dicrotic on account of the diastole being interrupted by the abortive systole.

The apex of the heart does not dilate, the aorta is empty because the ventricles do not dilate to receive the blood.

When given for a long time to the well or to the sick, death is produced by increasing the contractions causing the cavities to get smaller and smaller, and preventing the diastole or relaxation of the cavities.

Stoppage of the heart's action is due, not to paralysis, but to spasm, which occurs during the systole.

In poisoning, tannic acid is the antidote, while aconite is the best antagonistic when large doses have been given. Opium is useful when digitalis has been used for a long time, as the former produces relaxation of the heart.

From the foregoing we find the indications for the use of digitalis made clear; when the heart is weak, and only when the work required of that organ is less than the power to perform the same, digitalis is useful.

The drug can be given in dilated heart due to the relaxation of the muscles of that organ beyond their normal. In these cases the contractile power is lacking or weak, and the walls of the heart are unable to expel its contents owing to its relaxation. Digitalis here causes the necessary contraction and aids in emptying the cavities.

The drug is contraindicated in fatty heart and usually in cardiac hypertrophy, except, possibly, when associated with dilatation.

In dropsy, when it is due to or associated with an interference in the action of the kidneys, digitalis is good, as it is eliminated by the kidneys, and hence its diuretic qualities.

In pneumonia the danger we anticipate and encounter is a weak heart, and not, as a rule, a dilated heart, and in these cases digitalis is of no use. In this disease it may be indicated about the time of crisis, at which time the heart cannot drive the blood through the lungs, owing to the weakness of the walls of the right side of the heart, together with the obstruction in the lungs. Digitalis should not be given in this disease when the fever is high, as the heart is not dilated at this time (although it may be weak); but the organ relaxes when the fever suddenly falls, and hence in these cases digitalis does good. I speak of this because I think the use of digitalis has been, and is, abused in these cases, as it is used as a routine remedy.

A very important point which I wish to bring out, and which caused me to select digitalis as my subject, one that I find overlooked, not only by the student, but also by practitioners both of human and veterinary medicine, is that digitalis does not stimulate the heart in the same way that ammonia and alcohol do; but that, as I have already mentioned, it increases the contractile power of the heart-muscles, which power these muscles retain as long as the drug is given.

Another point is that the contractions in the arteries often remain after digitalis has been discontinued, and on this account obstruction in these vessels remains and interferes with the action on the heart. It is claimed that this arterial tension can be overcome by combining nitroglycerin with digitalis. Nitroglycerin dilates the arteries, the heart beats quickly, the contractions are powerful, but it does not interfere with the action of digitalis. Strophanthus may also be added, as it increases the power of the heart's action, but does not raise the tension in the arteries by interfering with their calibre. This combination is beneficial to overcome a weak heart due to arterial obstruction.

The action of digitalis is often increased by cold and by such drugs as ergot, belladonna, etc.

The preparations of the drug are the powder, abstract infusion, fluid extract, solid extract, and the tincture.

Of the powder, the horse takes from 10 to 40 grains, the dog 1 to 3 grains, the cow 30 to 60 grains.

The fluid extract of digitalis is probably the best form to administer it; the dose for the horse being 10 to 40 minims, the dog 1 to 3 minims, the cow $\frac{1}{2}$ to 1 drachm.

These doses are only approximate, as the so-called symptom medicines, of which digitalis is an example, have to be given gradually, increasing the dose till the physiological effect is produced.

The dose varies in individual cases, and as it is a very important point, the fact should not be overlooked in order to insure the proper results.

A very important feature in the administration of drugs is, *first*, the knowing what drug to prescribe; *second*, the kind of a case to which this particular drug applies; *third*, the quantity of the drug to be given in each case; *fourth*, the quality of the drug, and, *fifth*, the idiosyncrasies which may exist.

To sum up the actions and uses of digitalis:

1. It is a peculiar heart-stimulant (if the expression be allowed).

2. It is a useful diuretic in certain cases.

3. It increases the contractile power of the heart and arteries as well.

4. It does not stimulate the heart in the same way that alcohol and ammonia do.

5. Its action is aided, increased, and perfected in certain cases by the addition of nitroglycerin and strophanthus, and in other cases by cold, ergot, and belladonna.

6. It is a symptom medicine and should be pushed until its physiological effect is obtained.

7. It merely relieves certain symptoms, but does not cure disease.

8. It should not be given in pneumonia in a routine way, but only as the case requires, which usually is when the fever falls.

9. It is contraindicated in fatty heart and generally in cardiac hypertrophy.

10. The fluid extract is probably the best preparation to use.

11. The cinchona preparations, acetate of lead, sulphate and the tincture of the chloride of iron are chemically incompatible.

12. In cases of poisoning, use tannic acid, opium, aconite, or lobelia, and in some cases stimulants according to the indications.

